# Comparison of the ETView Single Lumen and Macintosh laryngoscopes for endotracheal intubation in an airway manikin with immobilized cervical spine by novice paramedics: A randomized crossover manikin trial

**DOI:** 10.1097/MD.0000000000005873

**Published:** 2017-04-21

**Authors:** Pawel Gawlowski, Jacek Smereka, Marcin Madziala, Barak Cohen, Kurt Ruetzler, Lukasz Szarpak

**Affiliations:** a Department of Emergency Medical Service, Wroclaw Medical University, Wroclaw, Poland; b Department of Emergency Medicine, Medical University of Warsaw, Poland; c Departments of General Anesthesiology and Outcomes Research, Anesthesiology Institute, Cleveland Clinic, Cleveland, OH.

**Keywords:** airway management, cervical spine stabilization, vivaSight SL

## Abstract

**Context:**

Management of the airway of a trauma victim is considered challenging. Various approaches have been described to achieve airway control in this setup; many of them include video-assited viewing of the larynx during intubation. ETView Single Lumen (SL) is a novice single-use endotracheal tube equiped with a video camera and a light source at its distal tip. Its use was previously described in seeral clinical and training setups.

**Objective:**

The aim was to evaluate the efficacy of the VivaSight SL compared with classic direct laryngoscopy performed with a Macintosh blade in a manikin-simulated trauma setup presenting various degrees of airway challenge when performed by inexperienced physicians.

**Design, Setting, Participants:**

This was prospective, randomized, crossover, manikin trial. After short training on the ETView system, 67 novice paramedics attempted to perform oral intubation using both standard direct laryngoscopy (MAC group) and the VivaSight SL endotracheal tube (ETView group) in a randomized order on manikins in 3 increasingly more difficult scenarios (simple intubation, cervical spine manual stabilization, and with cervical collar in place).

**Outcome Measure:**

Overall success rate, time to intubation, number of intubation attempts, laryngeal view grade, dental compression, and overall participant satisfaction were monitored.

**Results:**

Duration of intubation and number of attempts were significantly superior in the ETView group in the latter 2 more challenging scenarios. All other parameters showed superiority to the ETView group in all 3 scenarios.

**Conclusion:**

The VivaSight SL system performed better in a complex scenario of airway management of a trauma victim in need for cervical spine stabilization performed by novice caregivers compared to standard direct laryngoscopy and should be considered in this clinical setup.

## 1 Introduction

Securing the airway and subsequent ventilation to maintain oxygenation is a potentially life-saving procedure and therefore frequently indicated in the out-of-hospital setting.^[1,2]^ Airway management in the out-of-hospital emergency setting is especially challenging, as the emergency teams are frequently confronted with difficulties owing to facial trauma, pharyngeal obstruction, and limited access to the patient.^[3,4]^ In patients with a suspected neck trauma, cervical spine stabilization is indicated and furthermore complicates airway management.^[5–7]^ Although controversially discussed, securing the airway using an endotracheal tube is still considered the optimal technique, although this technique requires high level of personal experience and skills.^[8,9]^

Airway management in this challenging clinical setting was investigated by several studies in a wide range of devices and techniques, including direct laryngoscopy, fiberoptic intubation, blind oral/nasal intubation, and various supraglottic airway devices either as a primary device or as a bridge to blind or fiberoptic assisted intubation.^[10–16]^ In recent years, video-laryngoscopes were introduced into clinical practice with the ultimate goal to ease endotracheal intubation, especially in challenging clinical settings.^[17,18]^ Furthermore, there is increasing evidence that the use of videolaryngoscopes in patients with limited cervical spine mobility provides better visualization of the airway and finally facilitates endotracheal intubation.^[17–19]^

The ETView Single Lumen (SL) (VivaSight Ltd, Misgav, Israel) is a conventionally available single-lumen endotracheal, equipped with a video camera and a light source at its distal tip. The camera allows continuous visualization of the airway, which might be beneficial during intubation procedure.^[20–22]^ Furthermore, the ETView SL tube was described to be superior compared to direct laryngoscopy using a Macintosh blade in various resuscitation and trauma scenarios, by either paramedics, novice physicians, anesthesia residents, or certified anesthesiologists.^[23–25]^

The aim of this prospective cross-over study was to investigate whether endotracheal intubation by inexperienced paramedics using the ETView SL endotracheal tube is faster, and therefore clinically preferable, compared to direct laryngoscopy in manikins with stabilized cervical spine.

## 2 Methods

After approval of the institutional review board of the Polish Society of Disaster Medicine (Approval no: 17.06.2016) and obtaining written informed consent, 67 novice paramedics were enrolled. All paramedics had <1 year of experience and performed <10 endotracheal intubations in real patients. Furthermore, all paramedics had no previous experience with any videolaryngoscope or the ETView SL tube.

Based on pilot data from a previous study, the following assumptions were used to calculate the required sample size: The success rate of first endotracheal intubation attempt during uninterrupted chest compressions was 95% versus. 65% in the ETView and Macintosh groups, respectively.^[23]^ Using a paired 2-sided *t* test, accepting an α risk of ≤0.05, powered to 80%, 32 participants were required.

### 2.1 Study protocol

All paramedics underwent an initial training session lasting 30 minutes covering the relevant aspects of human anatomy, principles of endotracheal intubation, and detailed explanation and demonstration of the devices used in this study. Afterwards, the paramedics were allowed to familiarize themselves with both airway devices and were asked to perform at least one successful intubation with each device. All intubations were performed on a MegaCode Kelly advanced life support manikin (Laerdal Medical, Stavanger, Norway). This airway management trainer allows simulation of a normal airway and is widely used as an effective learning tool. The manikin was placed dorsal on the floor.

**Figure 1:**
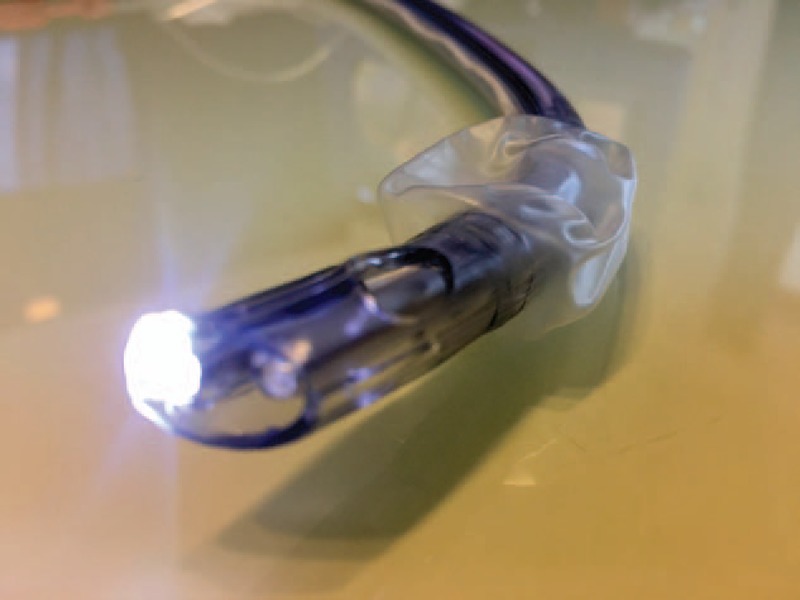
ETView SL witch integrated camera.

**Figure 2:**
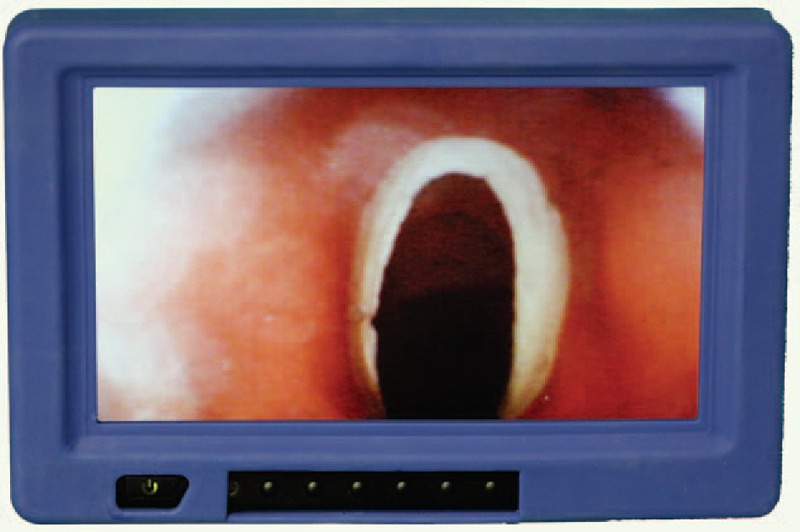
Vocal cords via dedicated monitor connected to ETView SL.

All paramedics were randomly assigned to 1 of 2 groups:

a) Direct laryngoscopy (Macintosh blade size 3), endotracheal intubation (Heine USA Ltd. Dover, NH) with a 7-mm I.D. tube
b) 7-mm I.D. ETView SL tube (VivaSight Ltd., Misgav, Israel; Fig. 1) connected to a dedicated monitor (Fig. 2).

Randomization was done by using ResearchRandomizer [www.randomizer.org] software.

The manikin and the tubes were lubricated. The tubes were equipped with a hockey-stick-shaped stylette, which was prepared by an experienced researcher. Paramedics were allowed to adjust the stylette as desired. After randomization, paramedics were asked to perform 3 intubations in 3 different subsequent scenarios:

1) Scenario A: normal airway (without cervical immobilization);
2) Scenario B: manual inline cervical immobilization, performed by an independent instructor
3) Scenario C: cervical immobilization using standard patriot cervical extraction collar (PatriotOessur Americas; Foothill Ranch, CA), which was applied to the manikin's neck by an independent instructor.

After paramedics completed the initial 3 scenarios, paramedics switched to the alternate intubation group and performed another 3 intubation scenarios in the same manner as described above.

All scenarios were limited to a maximum of 3 intubation attempts and each intubation attempt was limited to a maximum of 60 seconds. To avoid any teaching bias, all paramedics performed intubations alone and were not allowed to watch each other.

### 2.2 Measurements

Time to intubation (TTI), defined as the time from picking up the airway device until first successful ventilation of the lungs, served as our primary outcome. Additional secondary outcomes were time from picking up the device until visualization of the vocal cords (T1), time from picking up the airway device until successful intubation of the tube within the trachea (T2), subjective evaluation of ease of use using a visual analogue scale score ranging from 1 (extremely easy) to 10 (extremely difficult) and overall success rate of intubation. Vocal cord visualization was assessed by using Cormack & Lehane classification^[26]^ and severity of potential dental trauma, using the previously described grading scale,^[25]^ was performed after each intubation attempt. Finally, paramedics were asked which device they would prefer in a real-life emergency intubation setting.

### 2.3 Statistical analysis

Statistical analysis was performed using the Statistical version 12.0 for Windows (StatSoft, Tulsa, OK) software. A *P* value <0.05 was considered significant. Data are presented as number (percentage), mean ± standard deviation (SD), or median (interquartile range [IQR]), as appropriate. Nonparametric tests were used for the data that did not have a normal distribution. All statistical tests were 2-sided.

The Wilcoxon test for paired observations was used to compare the different times and to determine the statistical difference for each group. McNemar test was used to evaluate the differences in intubation success rates. Cormack-Lehane grade, ease of intubation score, severity of dental injury score, and preferred airway device were evaluated using the Stuart-Maxwell test.

## 3 Results

### 3.1 Study collective

**Figure 3:**
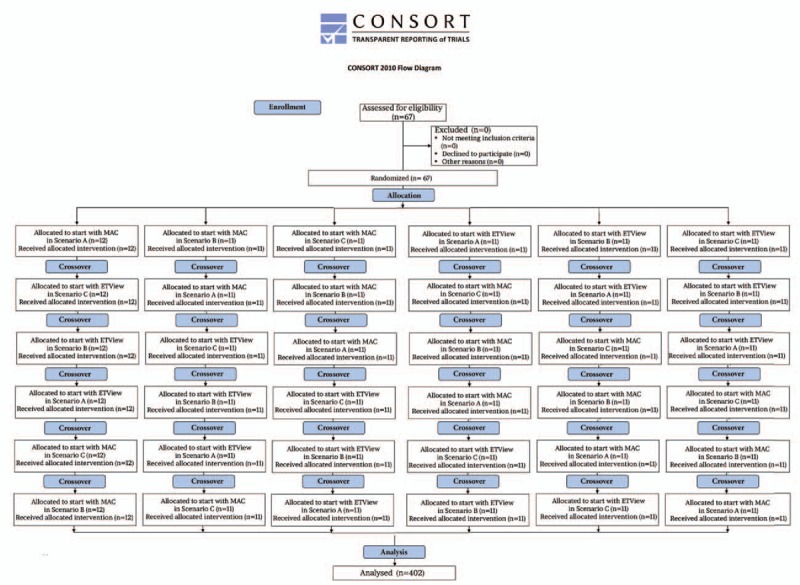
Randomization flow chart.

Sixty-seven novice paramedics were included in this study. The CONSORT diagram summarizing the flow of participants through the study is shown in Figure 3. The age of the paramedics was 26 ± 2 years, and the median (IQR) work experience was 0.5 (0.2–0.9) years.

### 3.2 Scenario A: normal airway without immobilization

**Figure 4:**
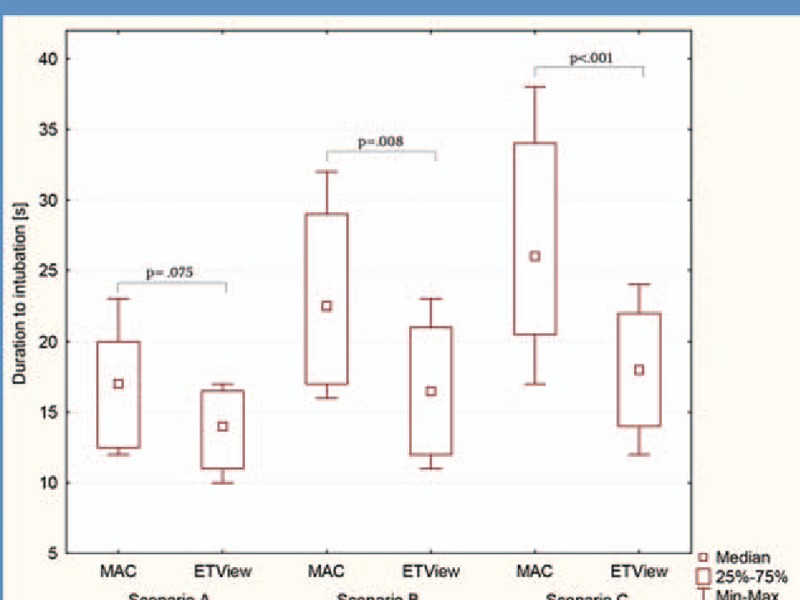
Duration of intubation using distinct devices in all research scenarios.

All paramedics were able to intubate the manikin with both devices, resulting in an overall success rate of 100%. In the ETView group, all paramedics were successful with the first intubation attempt. In direct laryngoscopy group, 90% of the paramedics were successful with the initial intubation attempt and the other 10% required a second intubation attempt (Table 1). Median time to intubate was comparable with both devices (Fig. 4).

**Table 1:**
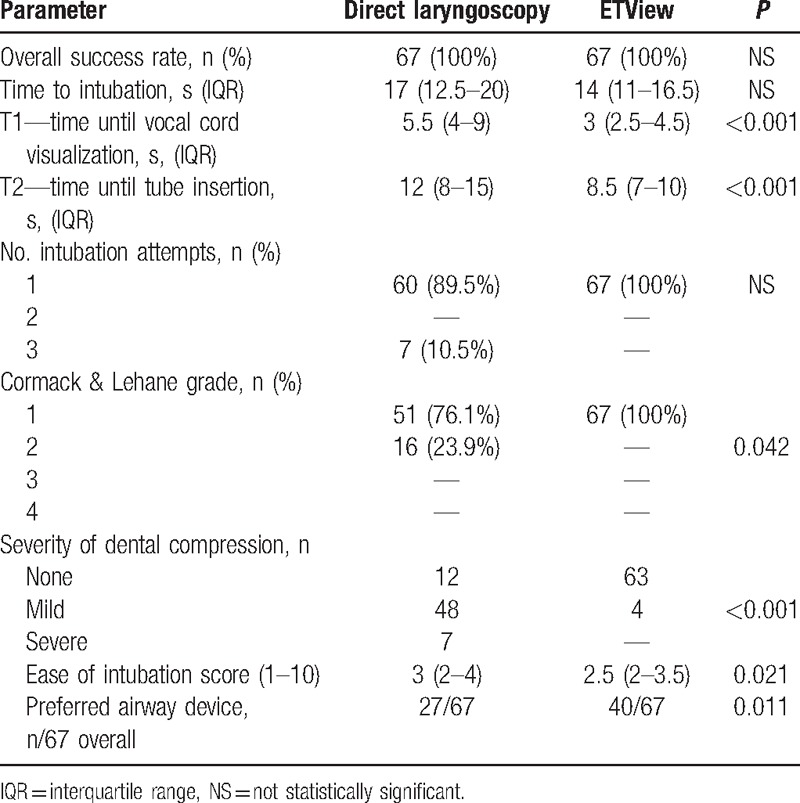
Normal airway without immobilization (Scenario A).

Difference in intubation attempts, Cormack & Lehane classification, ease of intubation, and assessment of preferred airway were not statistically significant.

### 3.3 Scenario B: manual inline cervical spine immobilization

Median time to intubate was faster in the in the ETView Group (16 vs. 22 seconds in direct laryngoscopy), but was statistically not significant (*P* = 0.008). The difference in time until vocal cord visualization and time until tube insertion, number of intubation attempts, and Cormack & Lehane Score were statistically not significant (Table 2).

**Table 2:**
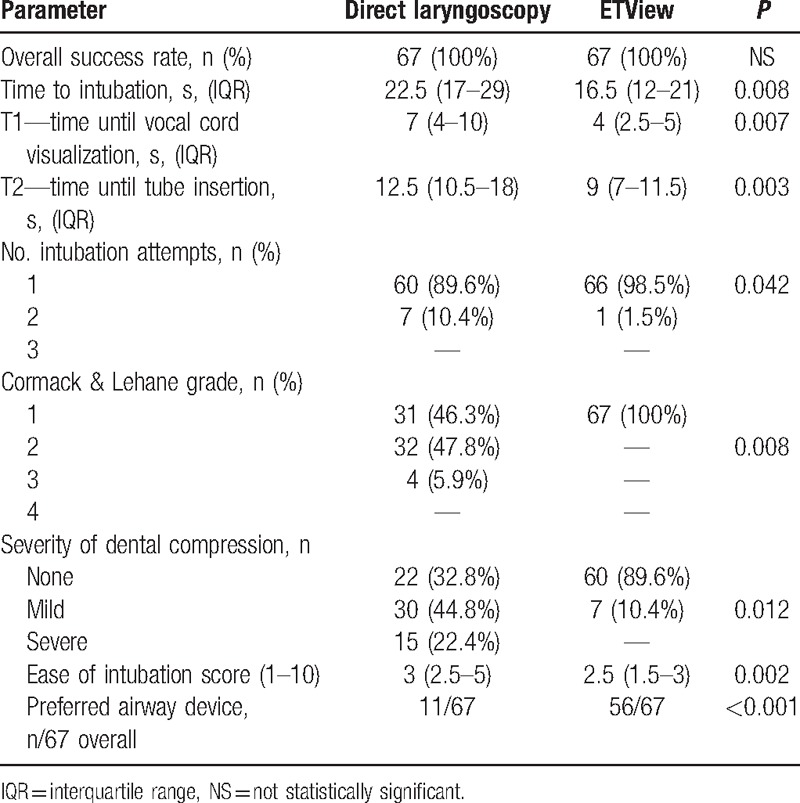
Manual inline cervical spine immobilization (Scenario B).

### 3.4 Scenario C: cervical immobilization using cervical extraction collar

Median time for intubation was significantly faster in the ETView group compared to the direct laryngoscopy group (18 vs. 26 seconds, *P* < 0.001) (Table 3). Intubation in the ETView group was also associated with significantly shorter time to glottis view, time to tube insertion. ETView intubation was also associated with better vocal cords visualization, number of intubation attempts, lower severity of dental compression, and easier intubation evaluation.

**Table 3:**
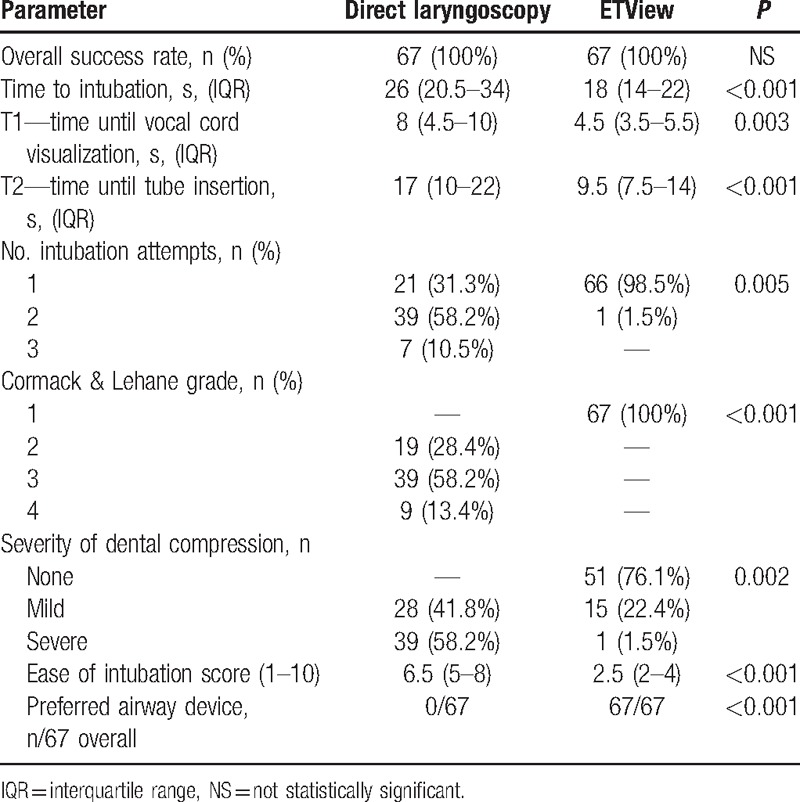
Cervical immobilization using cervical extraction collar (Scenario C).

## 4 Discussion

The most important finding of this study is that endotracheal intubation in manikins with immobilized cervical spine using the ETView SL tube was faster compared to direct laryngoscopy.

Several studies compared different modern technological solutions for the challenging airway management in resuscitation and trauma settings. In the current study, all participants were able to successfully intubate the manikin's trachea in all 3 scenarios in both groups. However, some findings do support the hypothesis that the ETView SL is superior compared to conventional direct laryngoscopy.

Time to intubation with both techniques was comparable in scenario A, the easiest setting. In the more difficult setting, when the cervical spine was immobilized, intubation was much faster using the ETView SL. Furthermore, the ETView provided much faster and much better glottis visualization. We therefore might conclude that faster and more important better glottis visualization enabled faster intubation, at least in our manikin study.

This finding is quite interesting, as there is a current debate, whether better glottis visualization improves time to intubation and decreases intubation attempts.^[17]^ However, in our study, intubation with ETView was faster in all 3 scenarios, compared to direct laryngoscopy. But once again, the difference of maximal 8 seconds is clinically trivial and might be clinically not relevant.

Success rate with the first intubation attempt might be clinically much more relevant. Although our study was not powered enough for this outcome, intubation using the ETView was associated with less intubation attempts. The paramedics were able to intubate the manikin with the first intubation attempt in the normal airway setting. In the manikin settings with immobilized cervical spine, only 1 of 67 paramedics failed with the first intubation attempt resulting in a first intubation attempt success rate of 98%, compared to 90% in Scenario B and 32% in Scenaroio C for the direct laryngoscopy.

Regarding overall preferred technique, most participants preferred the ETView instead of direct laryngoscopy. This might be based on the fact that paramedics were able to watch the intubation procedure.

As a limitation, this study was performed in manikins instead of real patients. Results of manikin-based studies are limited in interpretation with humans, but the use of the manikins avoids the ethical challenges of airway management in real patients, especially if performed by novice inexperienced operators. Furthermore, the use of the manikins allowed us to use a cross-over study design, which is statistically much powerful.

## 5 Conclusions

As a conclusion, this was the first study evaluation the ETView SL in manikins with immobilized cervical spine and the results were quite convincing. Intubation was much faster and required lesser intubation attempts compared to direct laryngoscopy.
